# Supplementary material for: The domesticated transposon protein L1TD1 associates with its ancestor L1 ORF1p to promote LINE-1 retrotransposition
Source: eLife. 2025 Mar 20;13:RP96850. doi: 10.7554/eLife.96850 (PMC11925450; doi:10.7554/eLife.96850)

FIGURE 1C

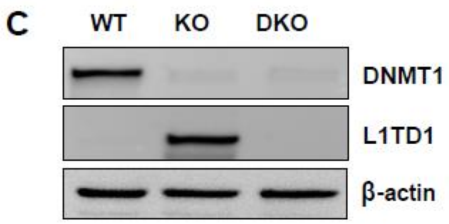

Prestained Protein Standards

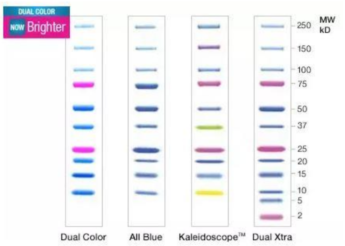

DNMT1

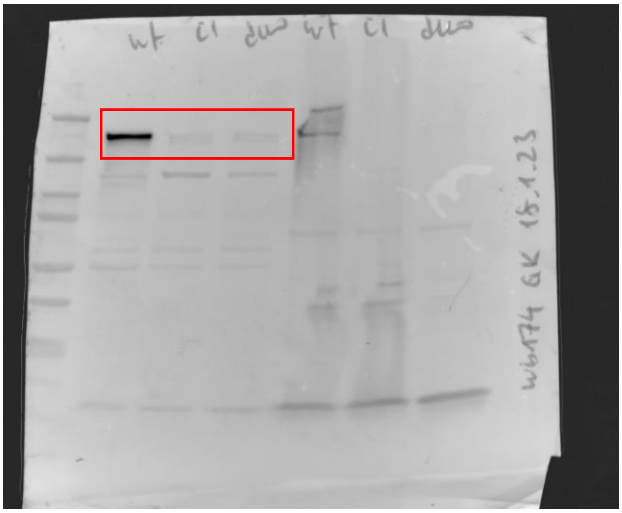

L1TD1

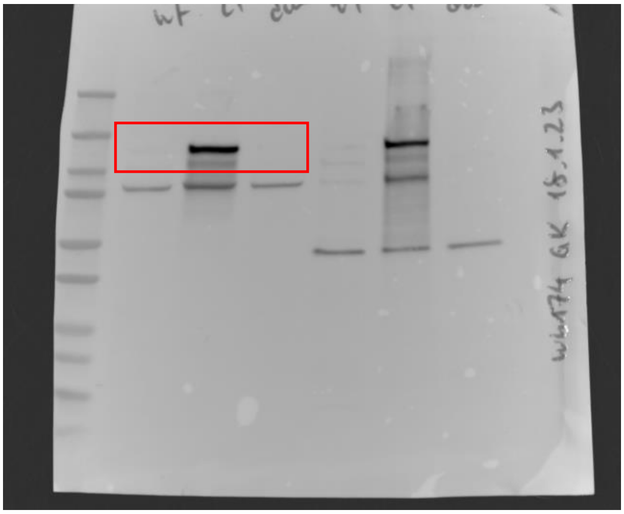

beta-actin

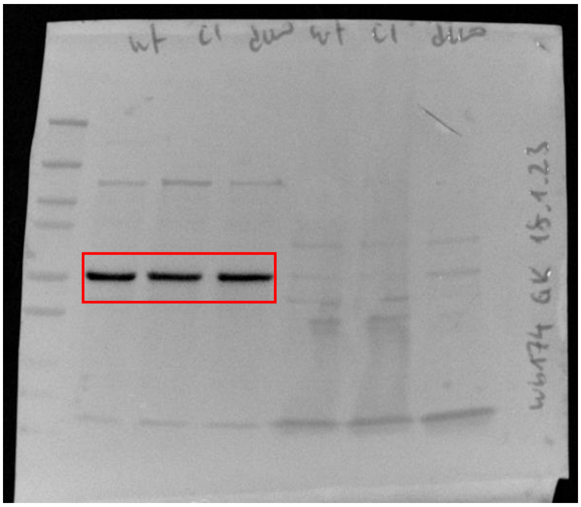

FIGURE 1D

D

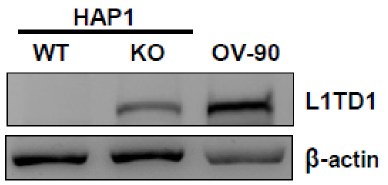

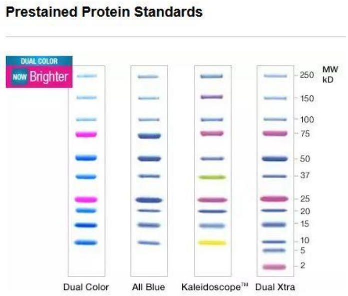

L1TD1

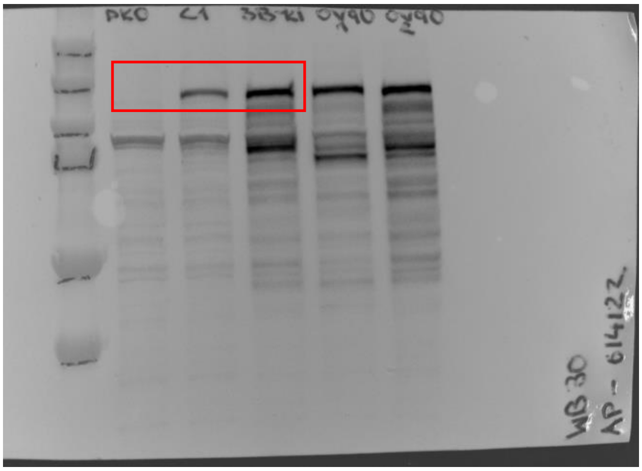

beta-actin

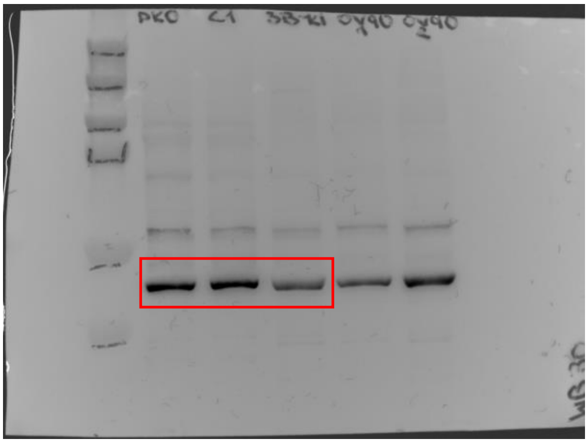

FIGURE 1H

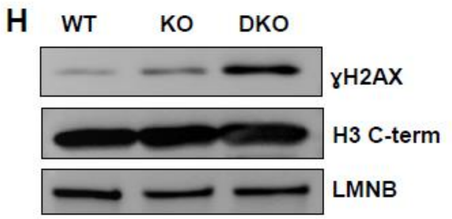

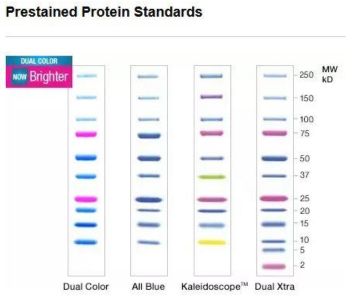

gamma-H2AX

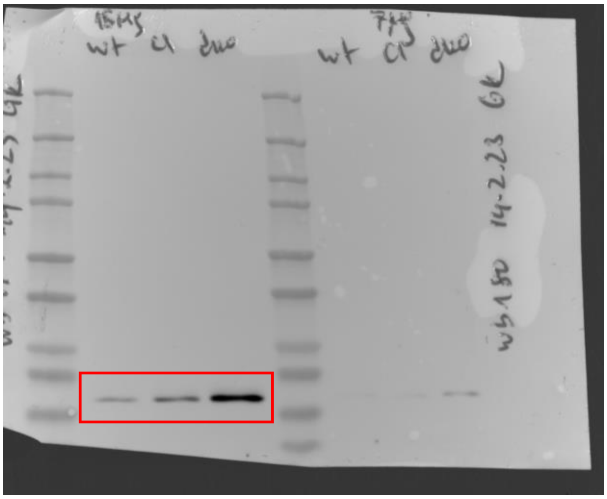

H3 C-term

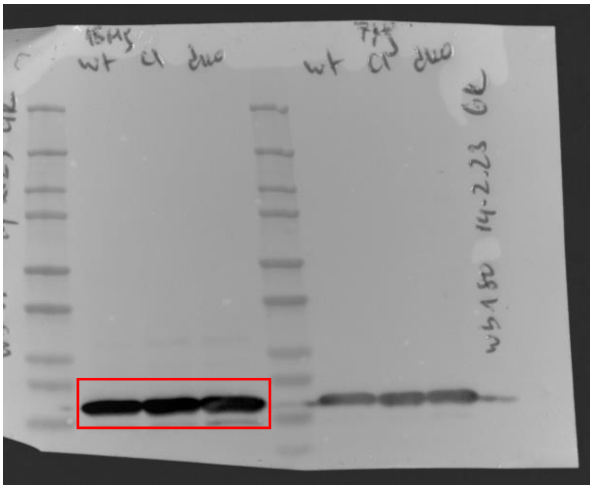

LAMIN B

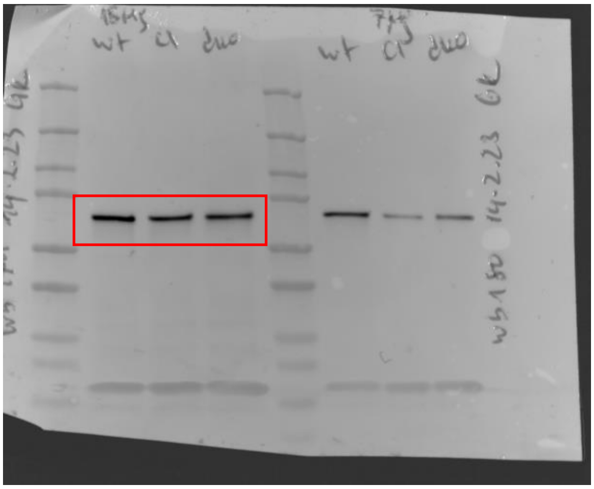

Supplement: Figure 1—source data 2. [file elife-96850-fig1-data2.pdf]
