# Supplementary figures and images for: The domesticated transposon protein L1TD1 associates with its ancestor L1 ORF1p to promote LINE-1 retrotransposition
Source: eLife. 2025 Mar 20;13:RP96850. doi: 10.7554/eLife.96850 (PMC11925450; doi:10.7554/eLife.96850)

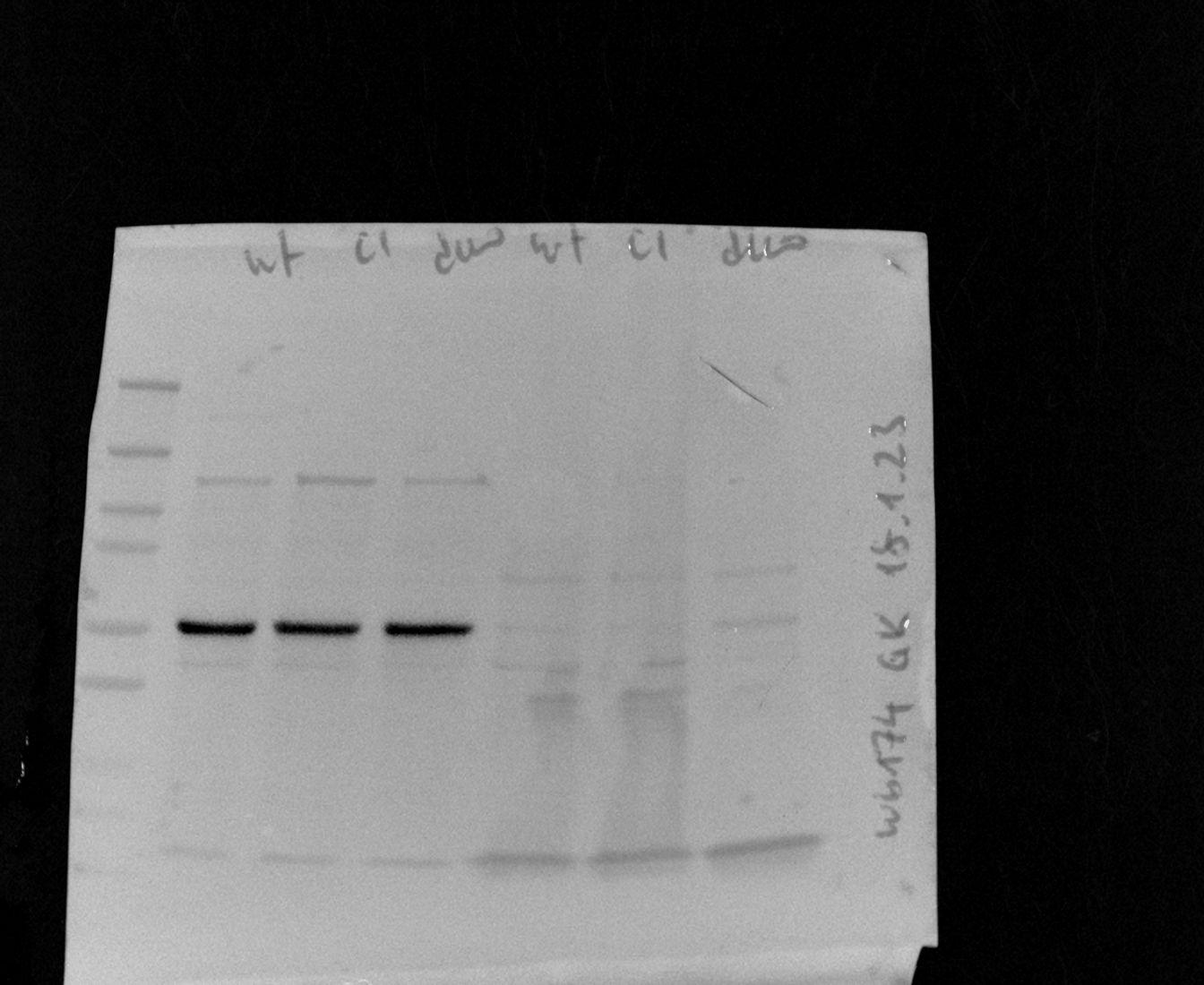

Supplement: Figure 1—source data 3. [file elife-96850-fig1-data3.zip › blots/Figure 1C beta actin.jpg]

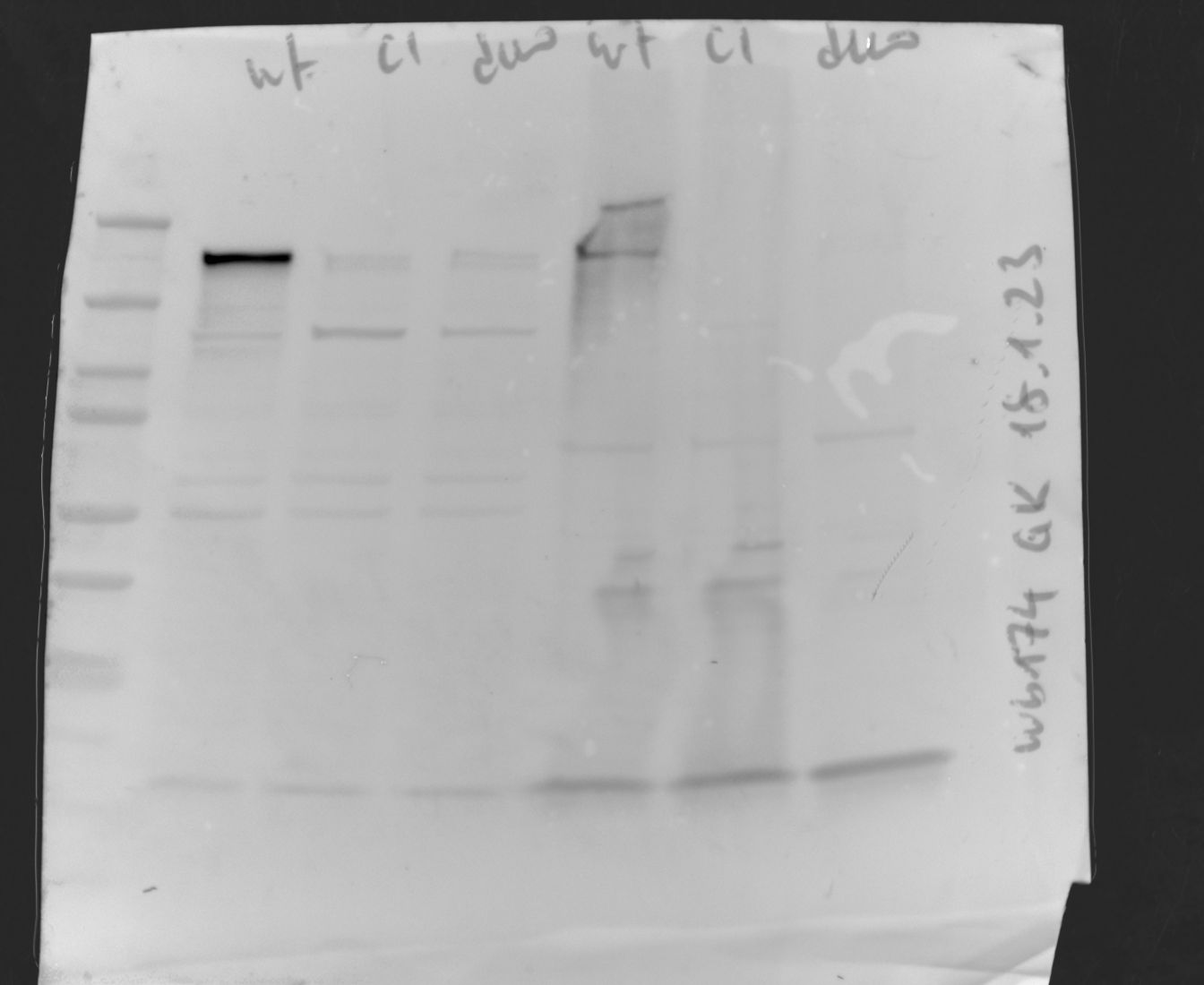

Supplement: Figure 1—source data 3. [file elife-96850-fig1-data3.zip › blots/Figure 1C DNMT1.jpg]

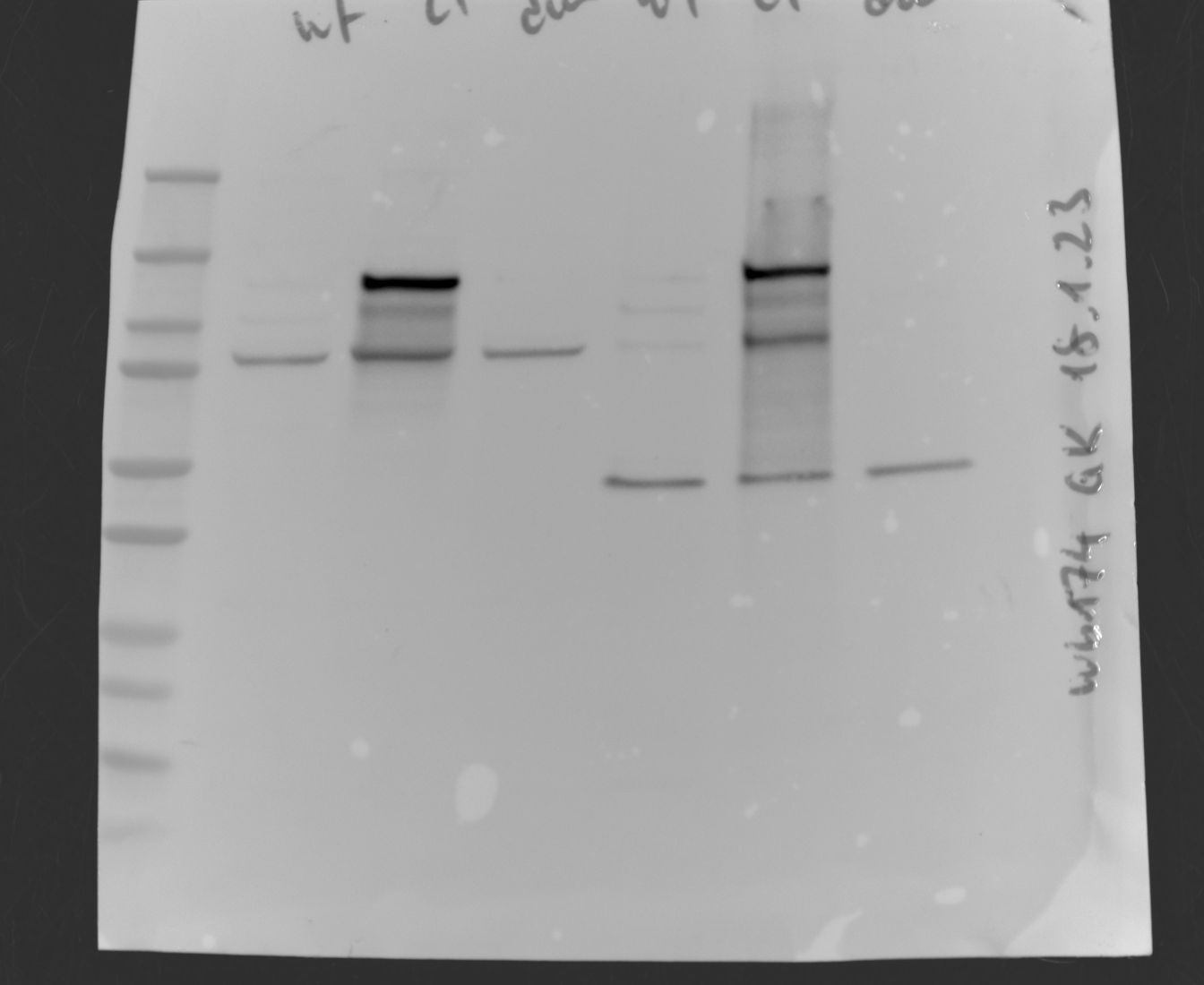

Supplement: Figure 1—source data 3. [file elife-96850-fig1-data3.zip › blots/Figure 1C L1TD1.jpg]

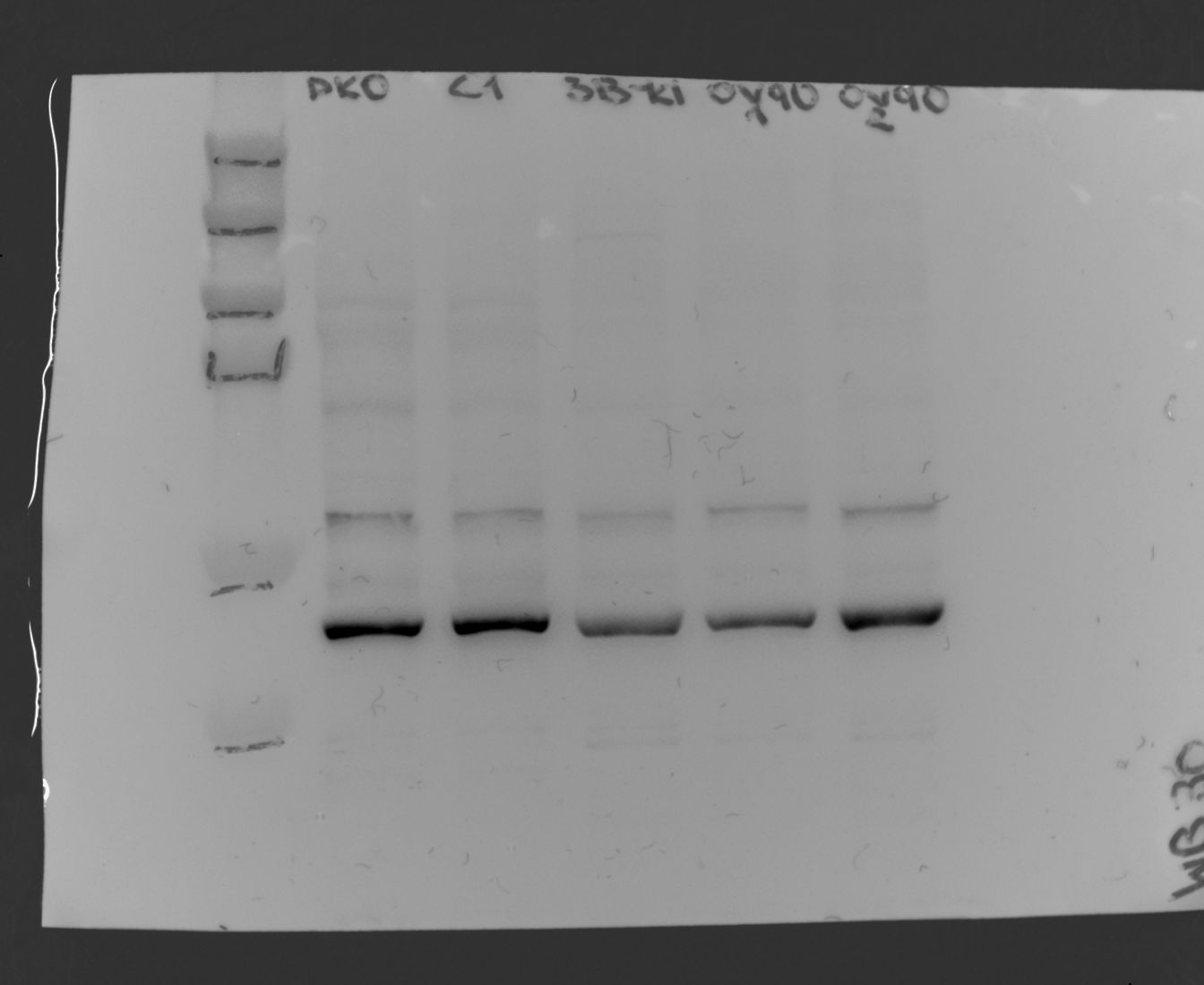

Supplement: Figure 1—source data 3. [file elife-96850-fig1-data3.zip › blots/Figure 1D beta actin.jpg]

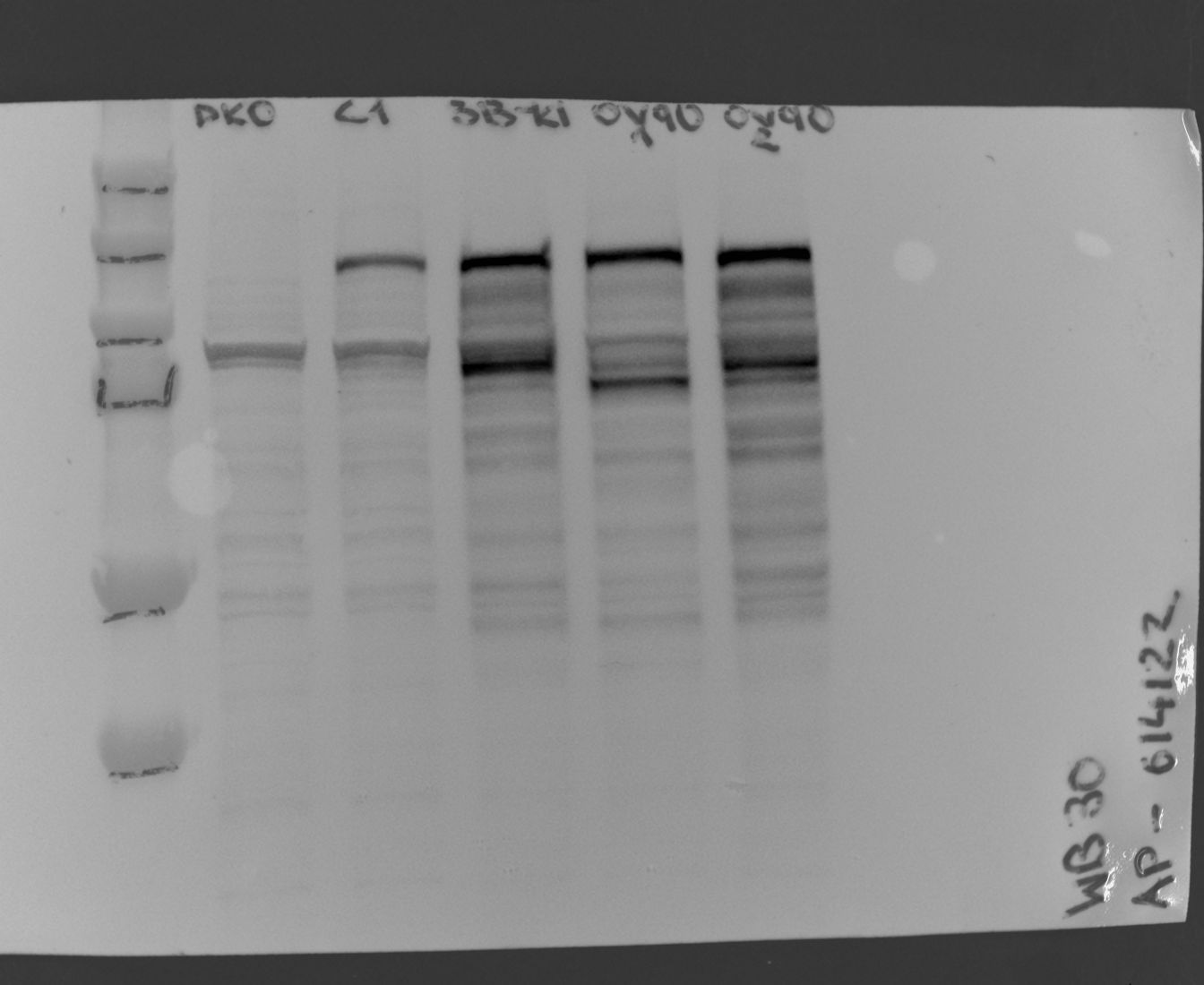

Supplement: Figure 1—source data 3. [file elife-96850-fig1-data3.zip › blots/Figure 1D L1TD1.jpg]

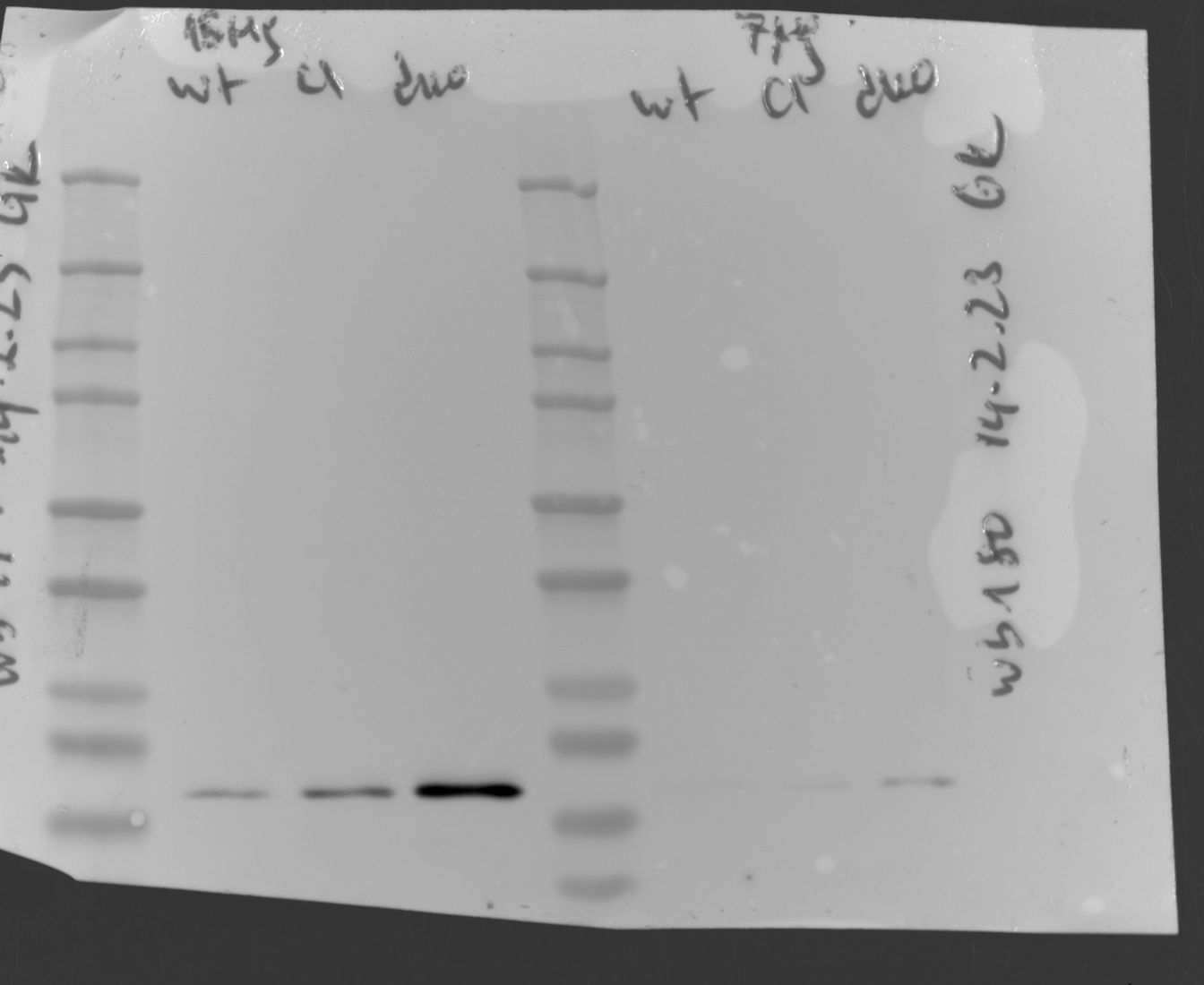

Supplement: Figure 1—source data 3. [file elife-96850-fig1-data3.zip › blots/Figure 1H gamma H2AX.jpg]

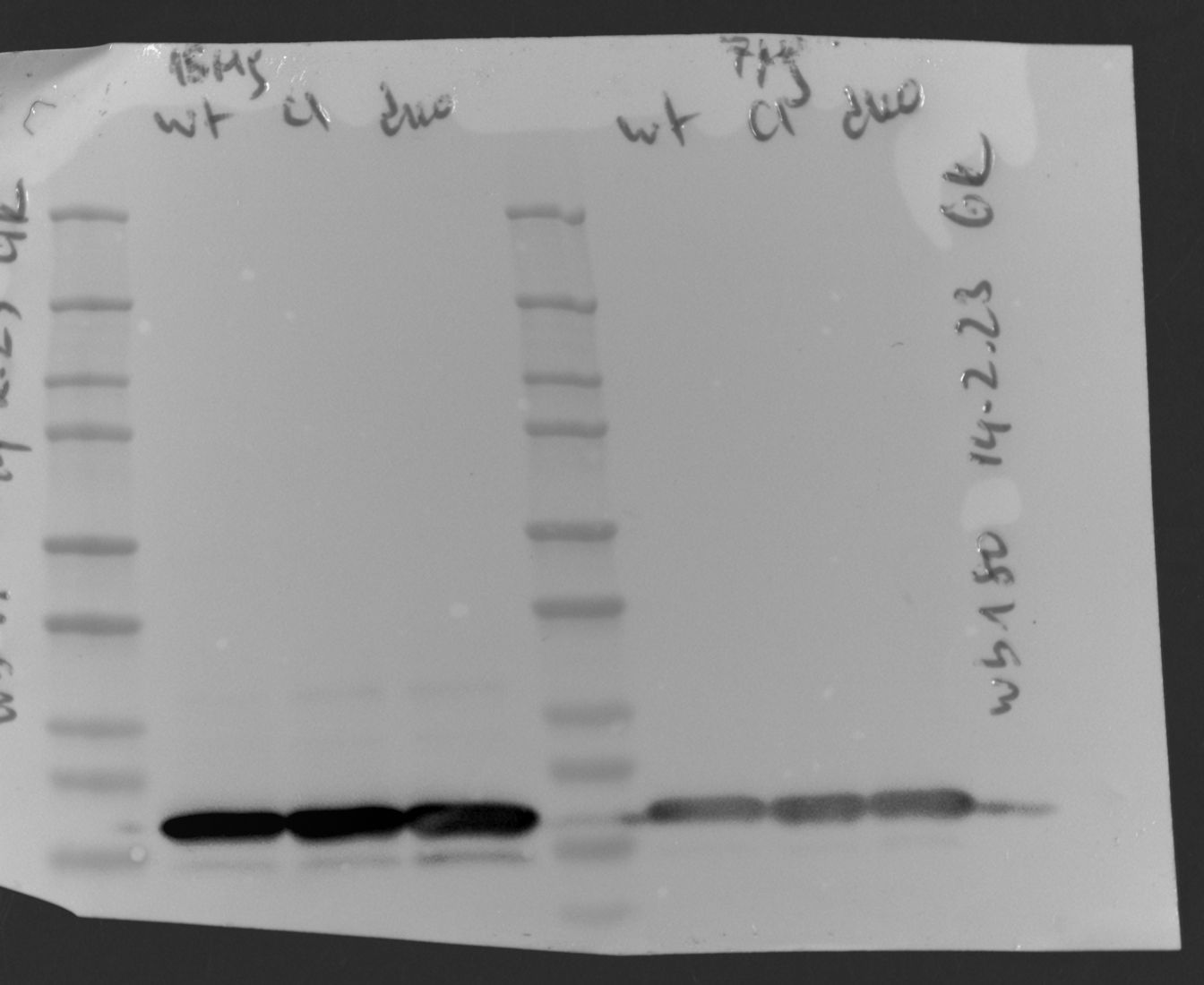

Supplement: Figure 1—source data 3. [file elife-96850-fig1-data3.zip › blots/Figure 1H H3 Cterm.jpg]

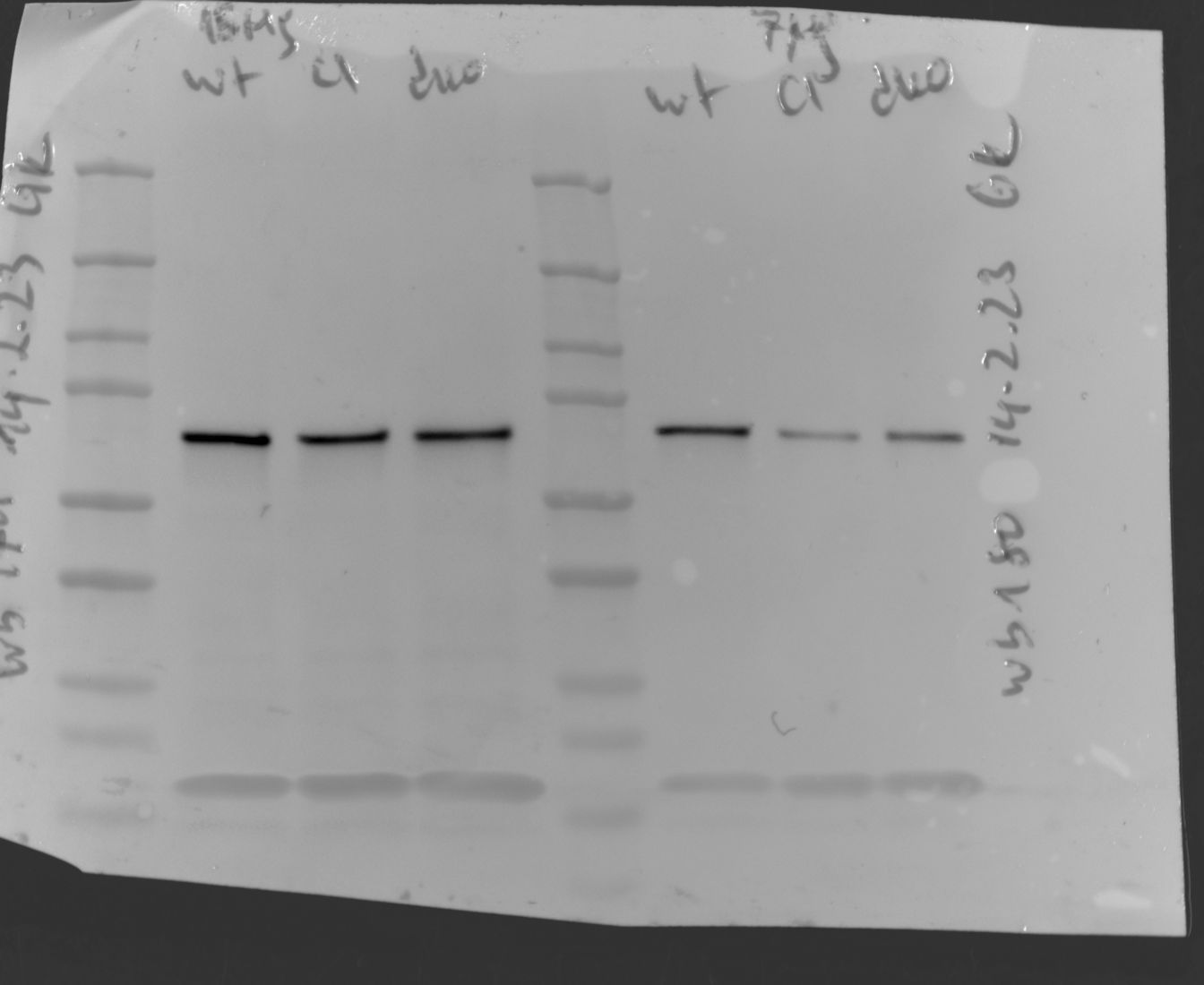

Supplement: Figure 1—source data 3. [file elife-96850-fig1-data3.zip › blots/Figure 1H Lamin B.jpg]

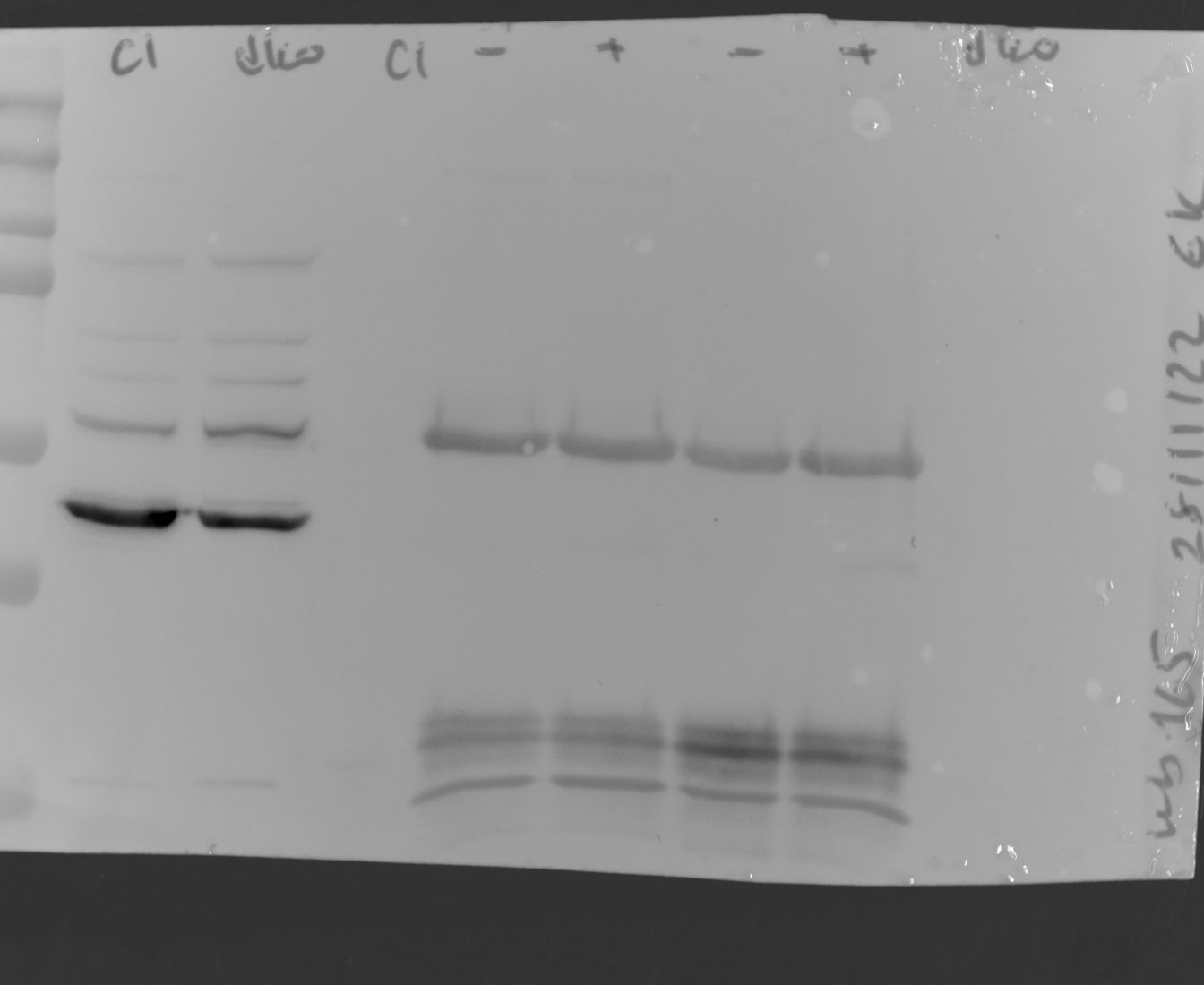

Supplement: Figure 3—source data 3. [file elife-96850-fig3-data3.zip › blots 2/Figure 3 S3A beta-actin.jpg]

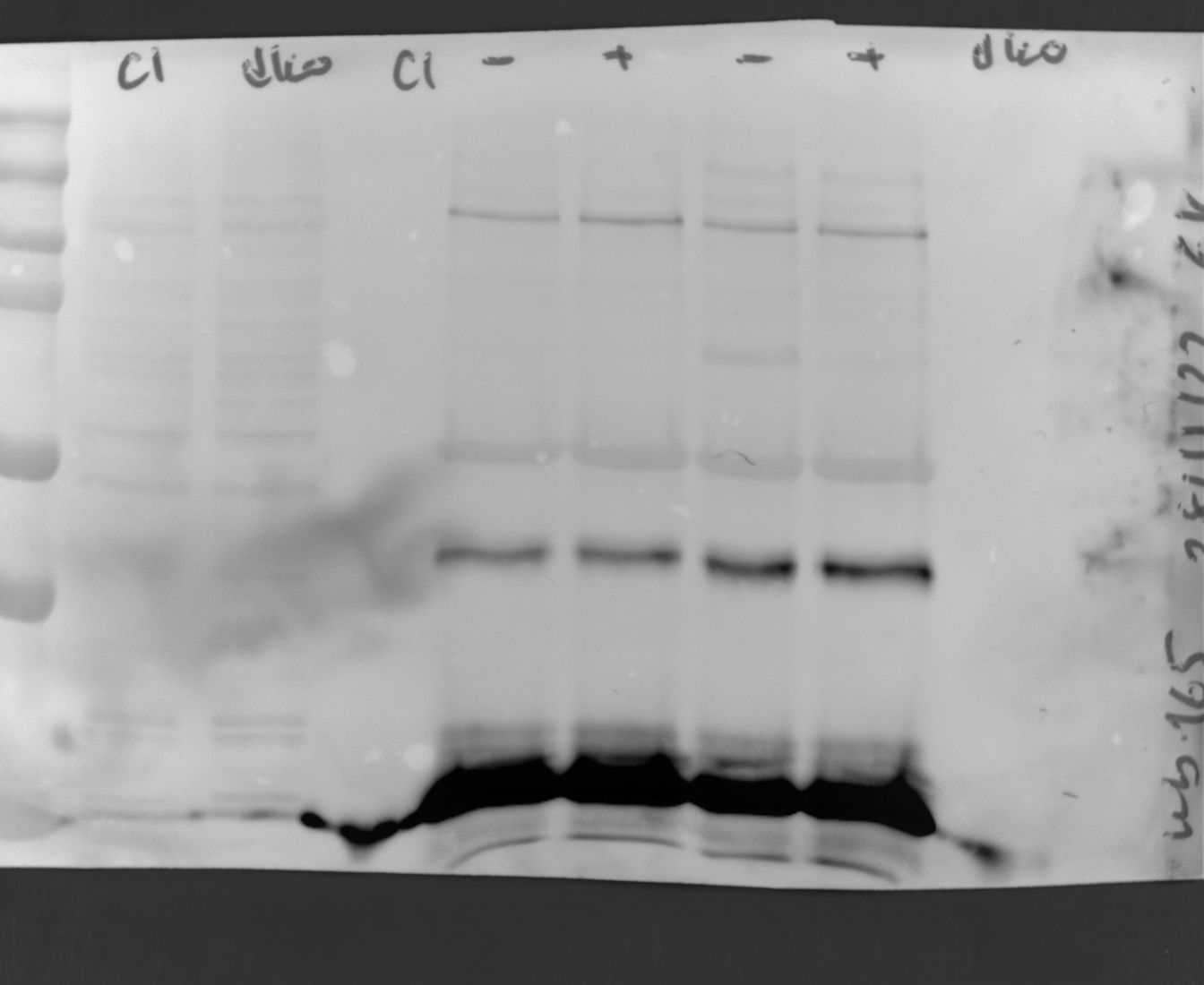

Supplement: Figure 3—source data 3. [file elife-96850-fig3-data3.zip › blots 2/Figure 3 S3A L1 ORF1p.jpg]

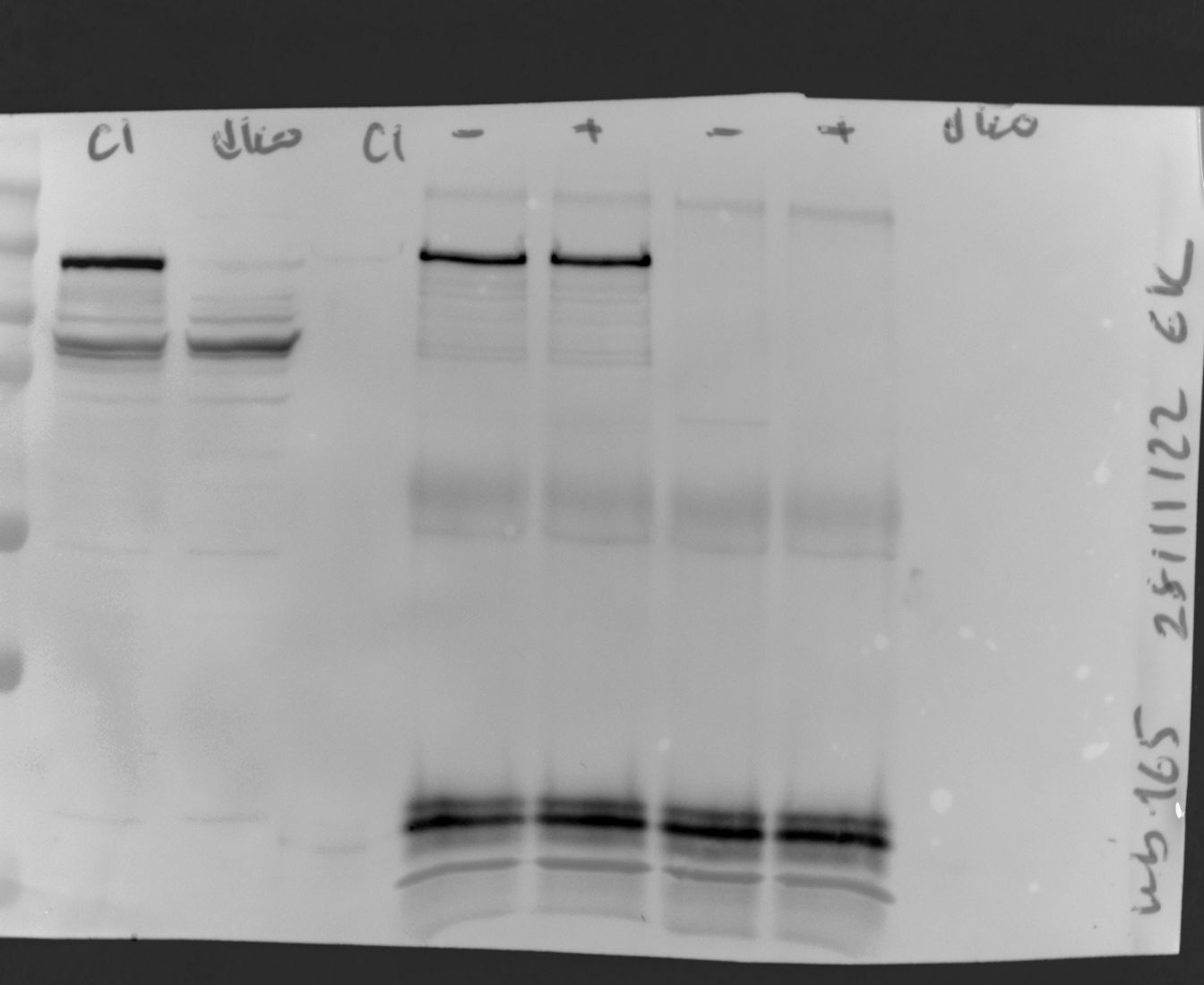

Supplement: Figure 3—source data 3. [file elife-96850-fig3-data3.zip › blots 2/Figure 3 S3A L1TD1.jpg]

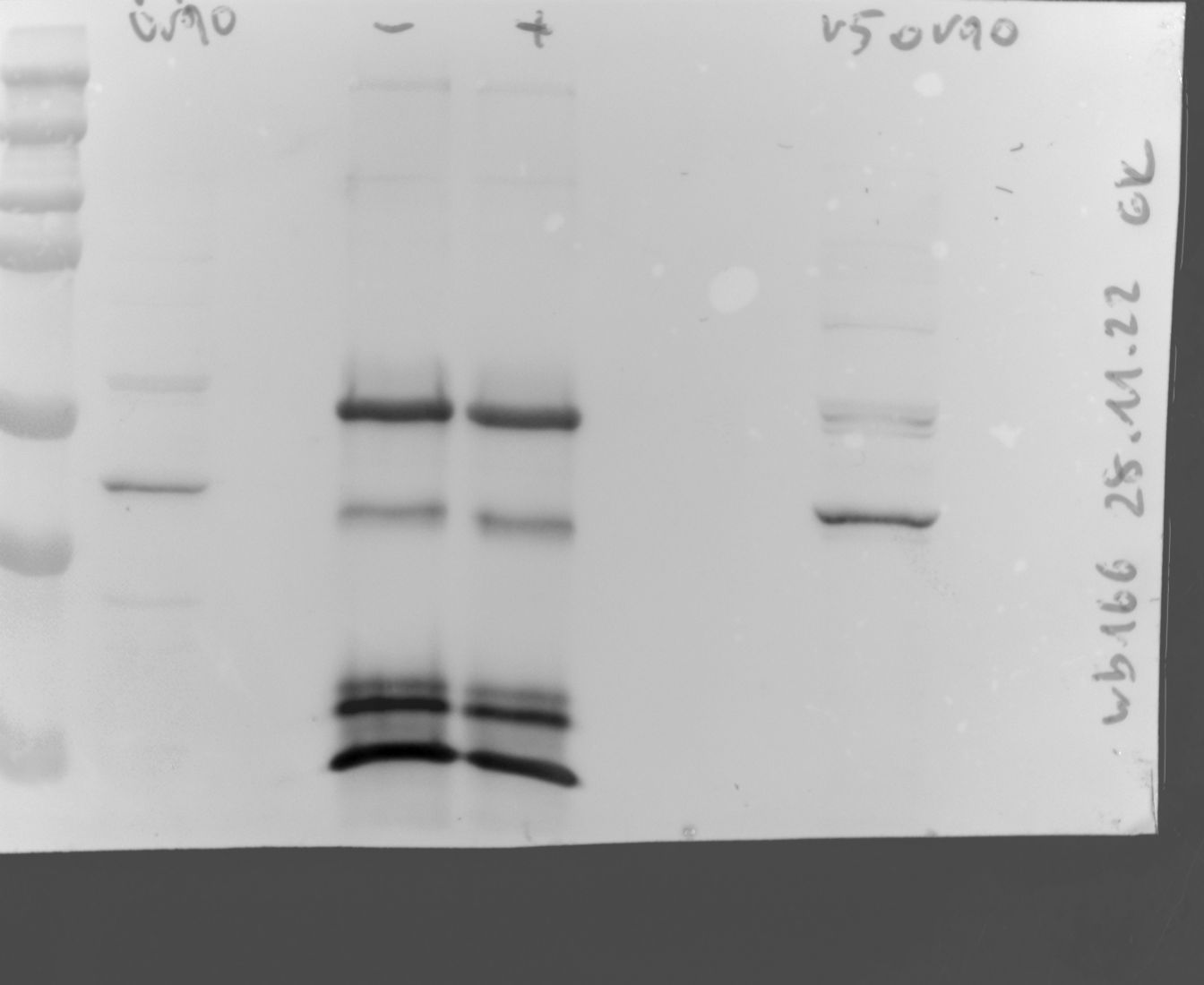

Supplement: Figure 3—source data 3. [file elife-96850-fig3-data3.zip › blots 2/Figure 3 S3B beta-actin.jpg]

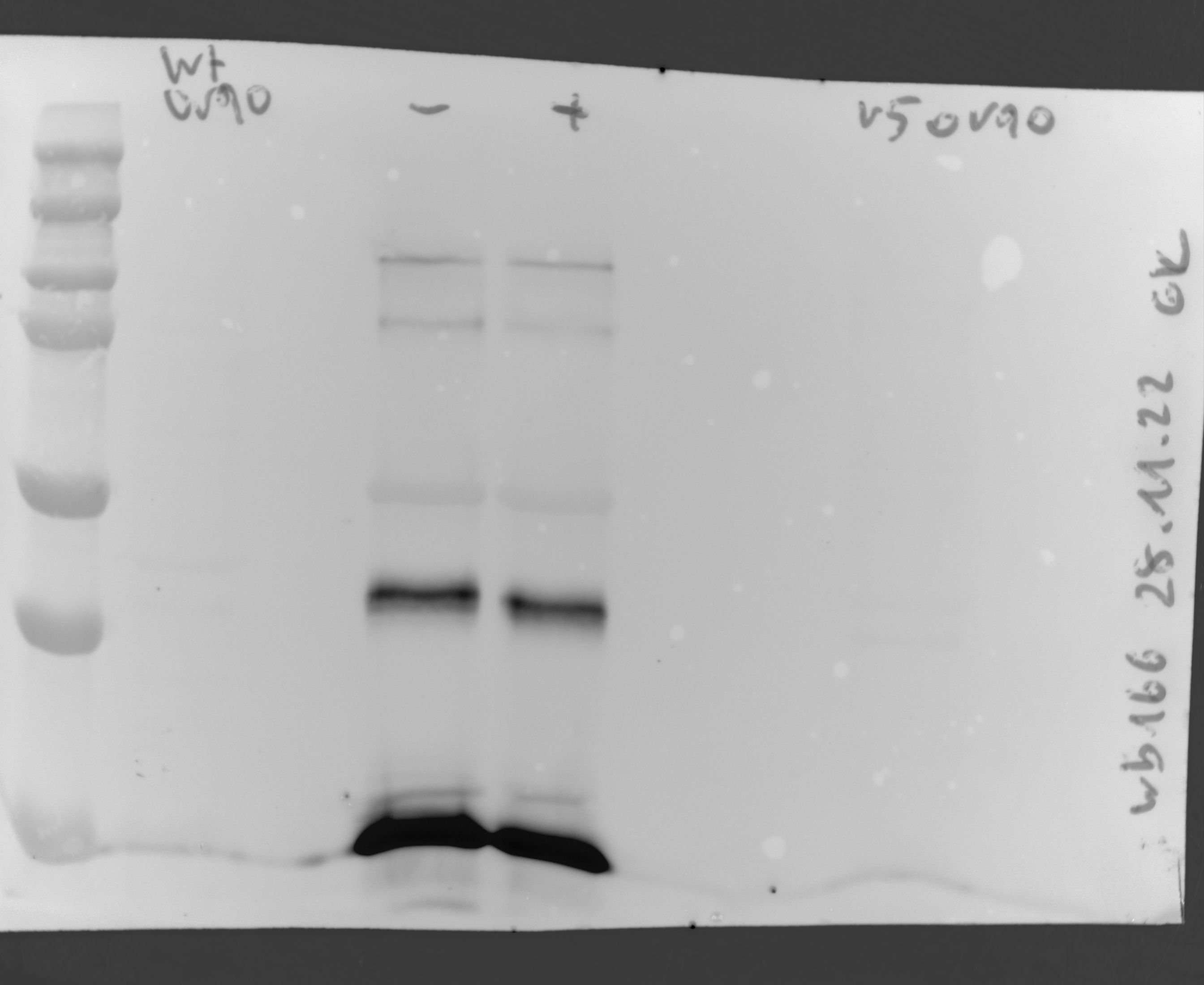

Supplement: Figure 3—source data 3. [file elife-96850-fig3-data3.zip › blots 2/Figure 3 S3B L1 ORF1p.jpg]

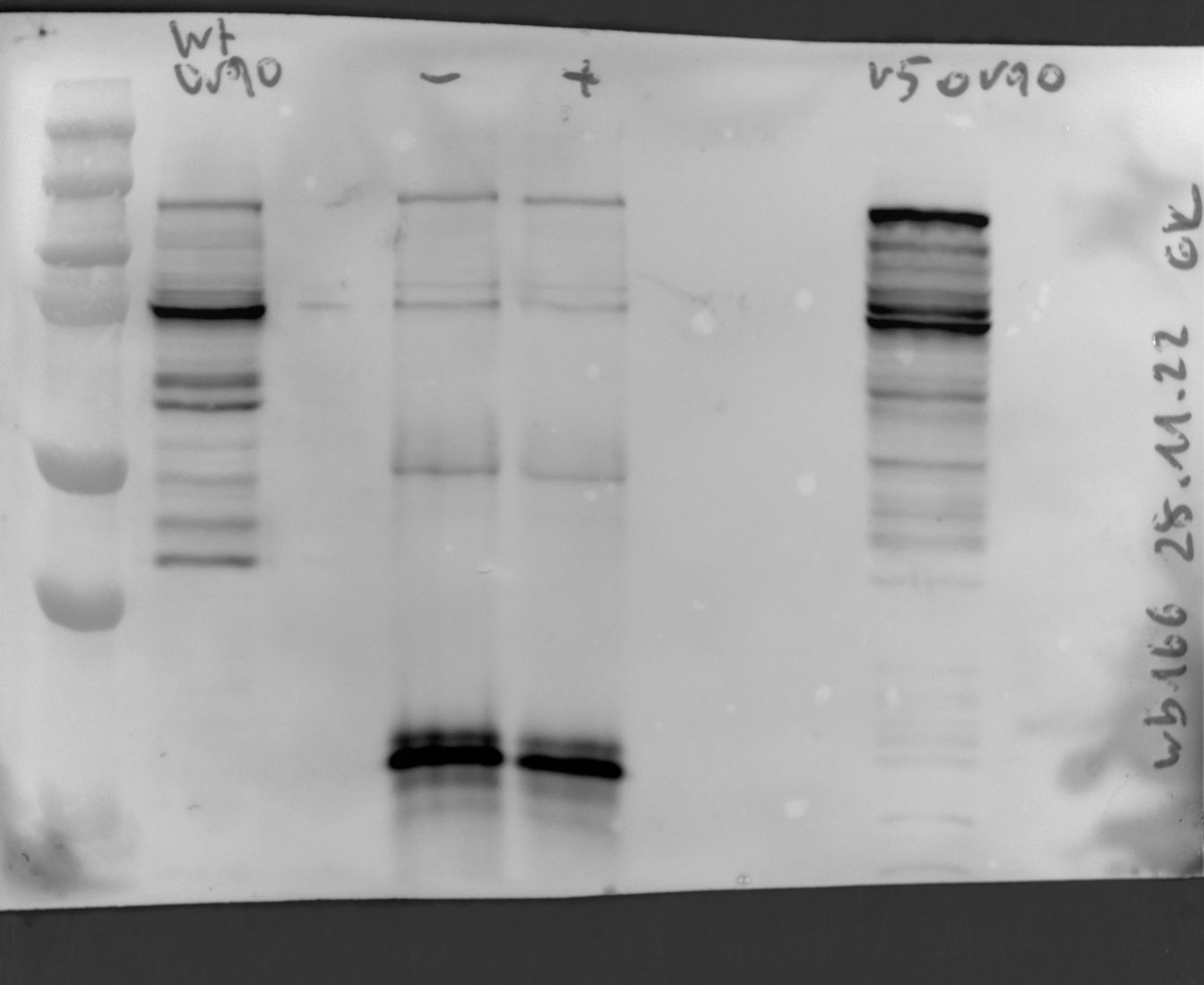

Supplement: Figure 3—source data 3. [file elife-96850-fig3-data3.zip › blots 2/Figure 3 S3B L1TD1.jpg]
