# Supplementary material for: The domesticated transposon protein L1TD1 associates with its ancestor L1 ORF1p to promote LINE-1 retrotransposition
Source: eLife. 2025 Mar 20;13:RP96850. doi: 10.7554/eLife.96850 (PMC11925450; doi:10.7554/eLife.96850)

C

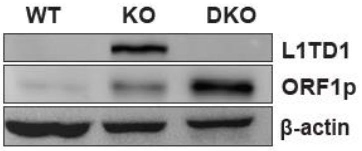

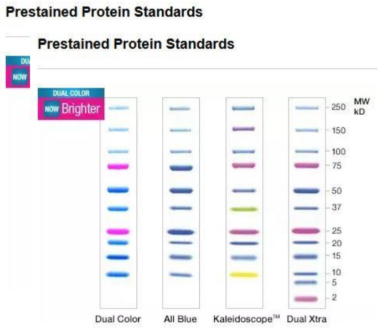

L1TD1

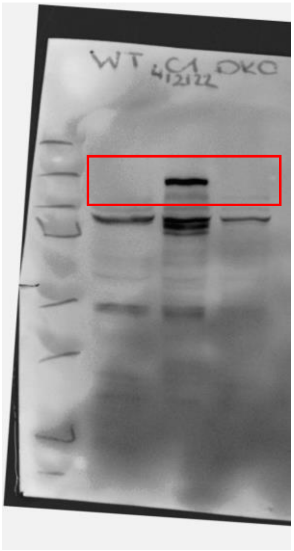

L1 ORF1p

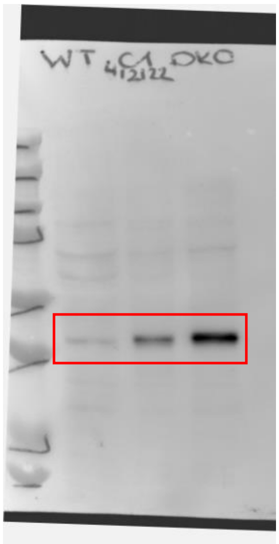

beta-actin

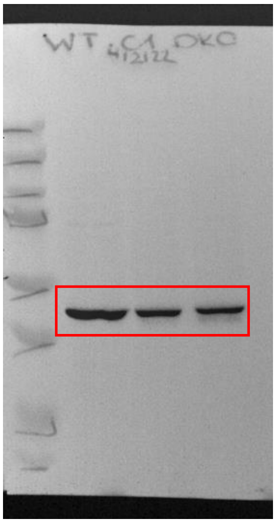

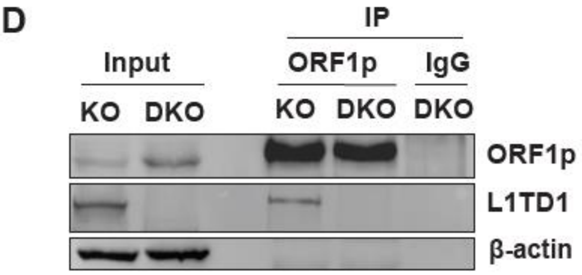

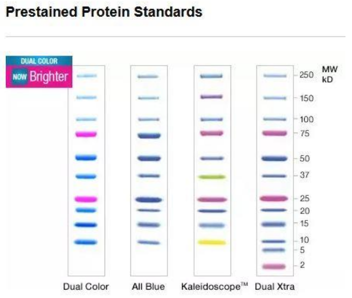

L1 ORF1p

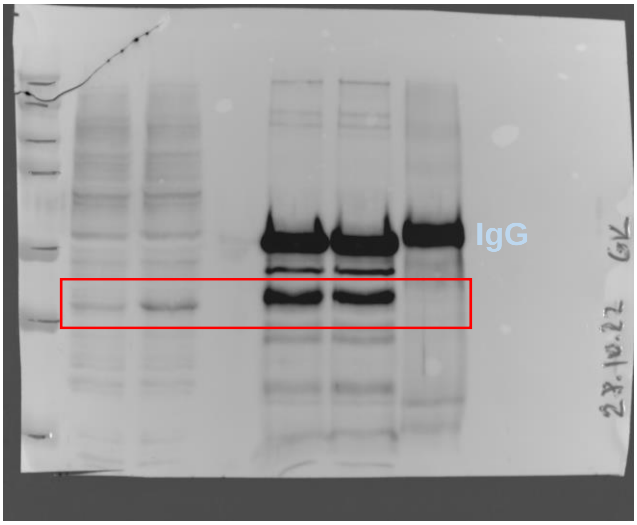

L1TD1

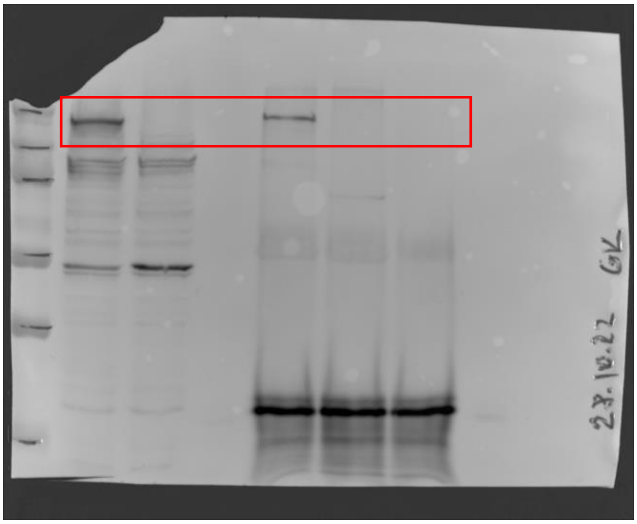

beta-actin

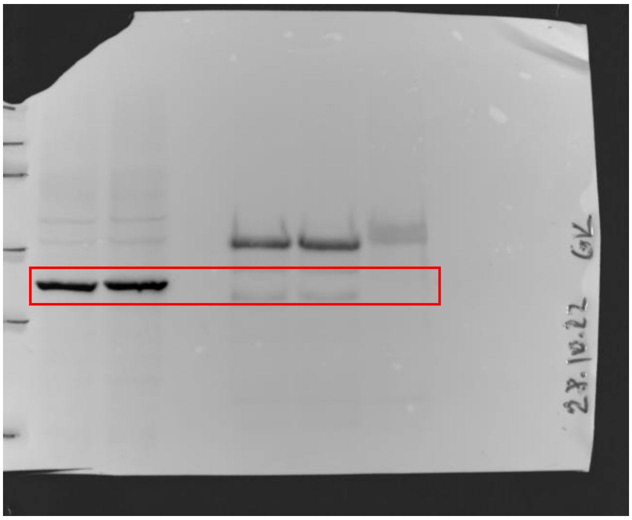

Supplement: Figure 3—source data 2. [file elife-96850-fig3-data2.pdf]
